# Understanding evolutionary and ecological dynamics using a continuum limit

**DOI:** 10.1002/ece3.7205

**Published:** 2021-05-01

**Authors:** Peter Czuppon, Arne Traulsen

**Affiliations:** ^1^ Institute of Ecology and Environmental Sciences Paris UPEC CNRS IRD INRA Sorbonne Université Paris France; ^2^ Centre Interdisciplinaire de Recherche en Biologie CNRS Collège de France PSL Research University Paris France; ^3^ Department of Evolutionary Theory Max Planck Institute for Evolutionary Biology Plön Germany

**Keywords:** continuum limit, diffusion approximation, extinction time, fixation probability, stationary distribution

## Abstract

Continuum limits in the form of stochastic differential equations are typically used in theoretical population genetics to account for genetic drift or more generally, inherent randomness of the model. In evolutionary game theory and theoretical ecology, however, this method is used less frequently to study demographic stochasticity. Here, we review the use of continuum limits in ecology and evolution. Starting with an individual‐based model, we derive a large population size limit, a (stochastic) differential equation which is called continuum limit. By example of the Wright–Fisher diffusion, we outline how to compute the stationary distribution, the fixation probability of a certain type, and the mean extinction time using the continuum limit. In the context of the logistic growth equation, we approximate the quasi‐stationary distribution in a finite population.

## Graphical abstract

We review the use and the derivation of continuum limits in the context of ecology and evolution and provide key analytic expressions of the fixation probability, the mean extinction time and the stationary distribution. By example, we compute these quantities for the Wright–Fisher process and the stationary distribution of the logistic growth equation.

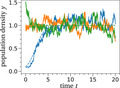

## 1 INTRODUCTION

The huge computational power available today allows researchers to develop individual‐based models of high complexity to explore dynamical processes in ecology and evolution. Here, we aim to make a link between these individual‐based descriptions and continuous models like (stochastic) differential equations that remain amenable to analysis. We review these techniques and apply them to some frequently used models in ecology and evolution.

In ecology, probably the most common description of population dynamics is the logistic growth equation (Verhulst, 1838). Its attractiveness draws from its simplicity. It has a globally attractive fixed point (when started with any nonzero population size), the carrying capacity of the population. This simplicity comes at the cost that biological observations like population size fluctuations or even extinction events are not captured by this deterministic model. To account for these stochastic effects, one needs to change to a stochastic differential equation which can be derived from individual‐based reactions (Champagnat et al., 2006). Stochastic differential equations consist of a deterministic and a stochastic term, the latter also often referred to as noise. In theoretical ecology, the effect of random environmental fluctuations on deterministic dynamics like the logistic equation has been studied in great detail (e.g., Allen et al., 1993; Schaffer et al., 1986). Demographic stochasticity, which arises from the inherent randomness of birth and death events of individuals, is often modeled in discrete‐time models (e.g., Melbourne & Hastings, 2008; Schreiber et al., 2018). Models including demographic stochasticity in continuous time are more scarce in ecology. Typically, these models are studied in the context of evolutionary ecology, for example to model co‐evolution in ecological communities (Dieckmann & Law, 1996). More generally, questions related to extinction and coexistence, transitions between different stable deterministic equilibria or the maintenance of quasi‐cycles can be studied within the framework of stochastic differential equations (Boettiger, 2018; Jeltsch et al., 2019). Here, we outline how to derive stochastic differential equations from an individual‐based model. We do this along similar lines as reviewed in Black and McKane (2012) but with a larger emphasis on the technical details.

In evolutionary game theory, the Moran process has become a popular model for stochastic dynamics in finite populations (Nowak et al., 2004). It is a model describing the dynamics of different alleles in a population of fixed size and overlapping generations. As this is a birth–death process, quantities like fixation probabilities, fixation or extinction times, and the stationary distribution can be calculated based on recursions (Allen, 2011; Goel & Richter‐Dyn, 1974; Karlin & Taylor, 1975; Traulsen & Hauert, 2009). A continuum approximation for quantities that are known exactly may thus make limited sense at first sight—but it can provide a very useful new perspective. Another important process in population genetics is the Wright–Fisher process—a model for allele frequency dynamics in a population of fixed size and nonoverlapping generations (Wright, 1931). It is more popular in population genetics but is also used in evolutionary game theory (e.g., Imhof & Nowak, 2006; Taylor & Maciejewski, 2012; Traulsen et al., 2006; Wakano & Lehmann, 2014). The Wright–Fisher process is mathematically more challenging to analyze exactly than the Moran process. Therefore, continuum approximations resulting in stochastic differential equations are used to compute typical quantities of interest such as the probability of fixation of a certain genotype or the mean time until this fixation event occurs (Crow & Kimura, 1970; Ewens, 2004). Additional to the derivation of the continuum limit, we also present how to compute these quantities. In evolutionary game theory, demographic stochasticity is particularly important for behaviors that only evolve in small populations, such as spite (Gardner & West, 2004; Nowak et al., 2004). Initially, stochastic replicator dynamics have been developed to address the problem of equilibrium selection in evolutionary games (Cabrales, 2000; Foster & Young, 1990; Fudenberg & Harris, 1992). With the more recent development to look at games with a larger number of possible strategies, an approach based on weak mutation which allows to reduce the analysis to pairwise comparison between strategies became popular (Fudenberg & Imhof, 2006; Wu et al., 2011). These methods are particularly useful when many strategies have to be taken into account (García & Traulsen, 2019; Rand & Nowak, 2011; Sigmund et al., 2010; Traulsen et al., 2012; Vasconcelos et al., 2017). However, while the focus on weak mutation allows an analysis that takes demographic noise into account, additional approaches are necessary when, for example, internal equilibria are present (Black et al., 2012; Vasconcelos et al., 2017). The stochastic tools discussed here may be a further step into that direction.

Even though similar in the questions they try to answer, evolutionary game theory and population genetics are developing in parallel, sometimes with little interaction between them. As this is partly arising from the different methods applied, here we aim to provide an introduction to the continuum limit for those less comfortable with these methods and hesitant to go into the extensive, more mathematical, literature. In summary, for the theoretical population geneticist with a probabilistic background, we provide a summary of some key results on stochastic differential equations; for the evolutionary game theorist, we give a new perspective on the derivations of results obtained when using discrete birth–death processes; and lastly, for the theoretical ecologist familiar with deterministic modeling, we outline how to derive and work with stochastic versions of classical ecological and evolutionary processes.

Since our goal is to illustrate how to apply a continuum limit to individual‐based descriptions of a biological process, the calculations and derivations below may remain vague where more mathematical theory is necessary. For a mathematically rigorous presentation of this topic, we refer to the lecture notes by Etheridge (2012) or the book by Ewens (2004). A more application‐oriented treatment of stochastic processes in biology can be found in the books by Lande et al. (2003), Otto and Day (2007), and Allen (2011).

## 2 EVOLUTIONARY AND ECOLOGICAL PROTO‐TYPE PROCESSES

We outline the derivation of continuum limits by application to exemplary processes from evolution and ecology, the Wright–Fisher and Moran process, and the logistic equation. By showing the explicit derivation in these examples, we provide the necessary tool set to derive continuum limits of more complex processes motivated by individual‐based models. In this section, we define the models by their microscopic descriptions, that is, we describe the model dynamics as viewed from an individual's perspective.

### 2.1 Wright–Fisher and Moran process

The two most popular processes to model (stochastic) evolutionary dynamics are the Wright–Fisher and the Moran process. While in the Wright–Fisher process generations are nonoverlapping and time is measured in discrete steps, generations in the Moran model are overlapping and measured in either discrete or continuous time. Originally, both processes describe the stochastic variation of allele frequencies due to finite population size effects referred to as *genetic drift*.

#### 2.1.1 Wright–Fisher model

One of the oldest population genetics model is the finite size Wright–Fisher process (Fisher, 1930; Wright, 1931). Given a population of constant size, it describes the change in frequencies of alleles in nonoverlapping generations over time, measured in (discrete) generations.

Classically, one considers a population of *N* individuals where each individual is of type *A* or *B*. The population is considered to be in its ecological equilibrium. The population size *N* is therefore constant over time. One interpretation of the dynamics is that every generation each individual chooses, independently of all other individuals, an ancestor from the previous generation and inherits its type. Under selection, the likelihood of drawing type *A* individuals increases (or decreases) which introduces a sampling bias. The probability for an offspring to have a parent of type *A*, conditional on *k* individuals being of type *A* in the parental generation, is

(1) $$p_{k}=\frac{(1+s)k}{(1+s)k+N‐k},$$

where $s\in R_{\geq 0}$ is the selective advantage of type *A*. The number of type *A* individuals in the next generation is then given by a binomial distribution with sample size *N* and success probability *p_k_*. Denoting the number of type *A* individuals in generation *n* by *X_n,_* we have

(2) $$P\left(X_{n+1}=j|X_{n}=k\right)=\left(\begin{array}{c} N\\ j \end{array}\right)p_{k}^{j}{\left(1‐p_{k}\right)}^{N‐j},\;0\leq k\leq N.$$

Unfortunately, the Wright–Fisher model, even though very illustrative, is difficult to study analytically. Through the developments in stochastic modeling in the last century, a lot of this new theory could be adopted to overcome this problem (e.g., Ewens, 2004; Kimura, 1983).

#### 2.1.2 Moran model

Another way to resolve the difficulties associated with the Wright–Fisher process is provided by the Moran process (Moran, 1958). The setup is the same as for the Wright–Fisher process (constant population size *N* with two types or – in population genetics – alleles *A* and *B*) with one exception: Time is not measured in generations but each change in the population configuration affects only one individual, the one that dies and gets replaced by an offspring of another randomly selected individual. Therefore, generations are overlapping, and time can be measured in discrete steps or continuously.

##### Discrete time

The Moran process in discrete time progresses as follows. Every time step, one individual is randomly chosen to reproduce and the offspring replaces a randomly chosen individual among the remaining *N* − 1 individuals (sometimes, the replacement mechanism is not restricted to the remaining individuals but also includes the parent). Therefore, in a population with *k* type‐*A* individuals, the probability that one of these replaces a type‐*B* individual is given by

(3) $$T^{k+}=p_{k}\frac{N‐k}{N‐1},\;\text{for}\;0\leq k\leq N\;\left(\text{sometimes}\;T^{k+}=p_{k}\frac{N‐k}{N}\right)$$

with *p_k_* as defined in Equation 1. Analogously, the probability for the number of type‐*A* individuals to decrease from *k* to *k* − 1 reads

(4) $$T^{k‐}=\left(1‐p_{k}\right)\frac{k}{N‐1},\;\text{for}\;0\leq k\leq N.$$

We have implemented selection on the reproduction step, but it could also affect the replacement step. In a nonspatial setting, as considered here, this leads to the same transition probabilities. However, the Moran model can also be studied on a graph which aims to model spatial structure. In that case, the order of reproduction and replacement, and which of these steps is affected by selection, matters and can potentially give rise to different evolutionary dynamics (Kaveh et al., 2015; Lieberman et al., 2005). We note further that without selection (*s* = 0) we have *p_k_* = *k*/*N*, that is, the increase and decrease probabilities are equal for any choice of *k*. Dynamics with this property are called *neutral*.

##### Continuous time

The same dynamics (albeit on a different time scale) are obtained by assuming that each pair of individuals is associated with a random exponentially distributed time (also described as exponential clocks). The next pair to update their types is determined by the smallest random time (or the clock that rings first). At these updating times, one of the two individuals is chosen to reproduce, the offspring replacing the other individual of the pair. There is no standard choice when it comes to choosing the rate of these exponential times.

Both formulations of the Moran process are Markov chains, either in discrete or continuous time, with the special property of having jumps of ±1 only. These processes are called *birth–death processes*. The theory of these is well developed, see for example the books of Karlin and Taylor (1975, 1981), Gardiner (2004), or Allen (2011), so that the dynamics of Moran processes are often amenable to analysis (typically by solutions of recursion equations).

To sum up the introduction to these two evolutionary models, the difference between the Wright–Fisher model and the Moran model is the progression of populations in time. In the Wright–Fisher process, generations are nonoverlapping, that is, all individuals update their type at the same time. Therefore, the distribution of types in the offspring generation is binomial. In contrast, generations are overlapping in the Moran model and the dynamics are described by a birth–death process since only one individual is updated at a time.

### 2.2 Logistic growth

In ecology, one is typically interested in population sizes or densities rather than allele frequencies. The simplest population growth model is that of exponential growth. Obviously, a population cannot grow exponentially forever. Its growth will be limited at some point, for example due to spatial constraints or resource depletion. This form of density regulation suffices to stabilize a population around its carrying capacity, the positive population size at which in the deterministic process the growth rate equals zero.

Here, we give the mechanistic basis that could potentially describe such a process. We denote a single individual of the population by *Y*. The birth and death processes can then be written as

(5) $$\begin{array}{cc} Y\overset{\beta }{⟶}Y+Y, & \text{birth};\\ Y\overset{\delta }{⟶}\emptyset , & \text{death}. \end{array}$$

The parameters *β* and *δ* correspond to the rates at which the two reactions happen, that is, each reaction corresponds to an exponential clock with rate either *β* or *δ*. For *β* > *δ*, the population grows to infinity (exponential growth), whereas for *β* <*δ*, it goes extinct.

Population regulation is achieved through a nonlinear term that is interpreted as an interaction between two individuals, for example, competition for space. The corresponding microscopic process is given by

(6) $$Y+Y\overset{\gamma /K}{⟶}Y,\;\text{competition}.$$

The parameter *γ* is referred to as the intraspecific competition coefficient, and *K* is a measure of the number of individuals at carrying capacity. The division by *K* in the competition rate is accounting for the probability of interaction of two individuals in a well‐mixed population where space is measured by the parameter *K* so that *Y*/*K* becomes a density (or rate of encountering an individual when randomly moving in space). For a more detailed derivation of these type of interaction rates, we refer to Anderson and Kurtz (2015).

The logistic process is, like the Moran process, a birth–death process. We will see in the next section that in the infinite population size limit (we let *K* tend to infinity), the mechanistic description above yields the logistic equation

(7) $$\frac{dy}{dt}=ry\left(1‐\frac{y}{c}\right),$$

where *r* = *β* − *δ* is the per‐capita growth rate, *c* = (*β* − *δ*)/*γ* is the rescaled carrying capacity, and *y* = *Y*/*K* is the density of the population.

## 3 INFINITE POPULATION SIZE LIMIT

The microscopic descriptions can be implemented by a stochastic simulation algorithm. Yet, the theoretical analysis of finite size populations can be challenging. A common technique to overcome this challenge is to consider a continuum approximation, that is, studying the limiting model for *N* (or *K*) to infinity. The limit is a (stochastic) differential equation of the form

(8) $$dx_{t}=\mu \left(x_{t}\right)dt+\sqrt{\sigma^{2}\left(x_{t}\right)}dW_{t},$$

where (*W_t_*)*_t_*_ ≥ 0_ is a standard Brownian motion. This equation describes the population dynamics, that is, the macroscopic evolution of a certain model. For a general introduction to stochastic differential equations, see for example the books by Karlin and Taylor (1981) and Gard (1988).

The term *μ*(*x_t_*) is called the infinitesimal mean, that is, the expected change of the stochastic process (*x_t_*)*_t _*_≥ 0_ in a very short (infinitesimal) time interval. It represents the deterministic dynamics of the process. The term *σ* ^2^ (*x_t_*) is called the infinitesimal variance, that is, the expected variation of the continuum limit in very small time steps. It quantifies the random fluctuations. In the case where *σ* ^2^ (*x*) is zero, the limit is deterministic and Equation 8 reduces to an ordinary differential equation.

One can formally show that the terms *μ*(*x*) and *σ* ^2^ (*x*) indeed correspond to the changes of the mean and the variance in infinitesimally small time steps if they are derived formally (Appendix 1). This allows us to compute them by the following identities:

(9) $$\begin{array}{c} \mu \left(x\right)=\lim_{\Delta t\to 0}\frac{1}{\Delta t}E\left[\left(x_{\Delta t}‐x_{0}\right)|x_{0}=x\right],\\ \sigma^{2}\left(x\right)=\lim_{\Delta t\to 0}\frac{1}{\Delta t}V\left[\left(x_{\Delta t}‐x_{0}\right)|x_{0}=x\right], \end{array}$$

where E and V denote the expectation and variance of the process *x_t_*.

A solution of a stochastic differential equation of the form in Equation 8 is called a *diffusion*. Another common representation of Equation 8 is the following integral equation

(10) $$x_{t}=\begin{array}{c} \int_{0}^{t} \end{array}\mu (x_{s})ds+\begin{array}{c} \int_{0}^{t} \end{array}\sigma \left(x_{s}\right)dW_{s},$$

where the stochastic integral is interpreted in the sense of Itô. For a discussion of the different choices of stochastic integrals and their consequences in terms of modeling, we refer for example to Turelli (1977).

We now present how to derive Equation 8 for the three introduced models. The strategy is rather simple: Compute the infinitesimal mean and variance as given in Equation 9 for the individual‐based model.

### 3.1 Discrete‐time derivation: Wright–Fisher model

For the reason of illustration, we assume a Wright–Fisher model as outlined in Section 2.1 without selection, *s* = 0. We need to compute the expectations in Equation 9 using the probability distribution given in Equation 2 (with *s* = 0). Let us ignore the time step Δ*t* for the moment and simply compute the change in expectation and variance from one generation to the other. Writing *X_t_* for the number of individuals of type *A* and setting Δ*X_t_* = *X_t_* − *X_t_*_−1_, we find

(11) $$E\left[\Delta X_{t}|X_{t‐1}=k\right]=E\left[X_{t}|X_{t‐1}=k\right]‐k=N\frac{k}{N}‐k=0,$$

where we used that the number of individuals of a certain type in the next generation is binomially distributed. Analogously, the infinitesimal variance is

(12) $$\begin{array}{cc} E[{\left(\Delta X_{t}\right)}^{2}|X_{t‐1}=k] & =E\left[X_{t}^{2}\left|X_{t‐1}=k]‐2kE[X_{t}\right|X_{t‐1}=k\right]+k^{2}\\ & =V{\left[X_{t}\left|X_{t‐1}=k]+E[X_{t}\right|X_{t‐1}=k\right]}^{2}‐2k^{2}+k^{2}\\ & =N\frac{k}{N}\left(1‐\frac{k}{N}\right). \end{array}$$

It remains to account for the transition from discrete to continuous time. In this case, the natural choice is Δ*t* = *N* ^−1^. This can be seen by examining the infinitesimal variance. To obtain a limit different from zero or infinity in Equation 12 for *N* to infinity, we need to divide that equation by *N*. Comparing Equation 12 to the corresponding line in Equation 9, we set Δ*t* = *N* ^−1^. Replacing *k*/*N* by *x* and taking the limit *N* → ∞, we find the neutral Wright–Fisher diffusion for the allele frequency dynamics

(13) $$dx_{t}=\sqrt{x_{t}\left(1‐x_{t}\right)}dW_{t}.$$

This equation is called Wright–Fisher diffusion and describes the dynamics of a neutral allele due to genetic drift. In other words, the allele frequency behaves like a random walk in continuous time and space. A similar derivation as above can be done by including selection and mutation. The more lengthy computation is relegated to Appendix 2.

*Conclusion 1*: To derive a continuum limit of a finite population size model in discrete time, one computes the infinitesimal mean and variance as given in Equation 9 and rescales time so that the two quantities converge in a meaningful way, that is, do not tend to infinity.

### 3.2 Continuous‐time derivation: general case

In principle, the same methodology as above is applicable for the derivation of the continuum limit of a process measured in continuous time. However, in view of our subsequent analysis of the limit Equation 8, we will introduce a new tool, the *infinitesimal generator*.

The change in infinitesimal time of any continuous‐time Markov process (*x_t_*)*_t _*_≥ 0_ can be described by the infinitesimal generator, denoted (Ethier & Kurtz, 1986, chapter 1, eq. (1.10)). Intuitively, one can think of it as the derivative of the expectation (of an arbitrary function) of a stochastic process. Formally, it is defined by

(14) $$\left(Gf\right)\left(x\right)=\lim_{\Delta t\to 0}\left(\frac{E\;\left[f\left(x_{\Delta t}\right)|x_{0}=x\right]‐f\left(x\right)}{\Delta t}\right),$$

where $E\;[f\left(x_{\Delta t}\right)|x_{0}=x]$ denotes the conditional expectation of the stochastic process *f*(*x_t_*) at time Δ*t* given the initial value *x*_0_ = *x*. Here, *f* is an arbitrary function so that the limit is well‐defined. For example, applying G to *f*(*x*) = *x* describes the dynamics of the mean of *x_t_*, and for *f*(*x*) = *x* ^2^, we obtain the dynamics of the second moment of *x_t_*. From the first two moments, we can recover the variance, so that from Equation 14, one can derive the infinitesimal mean and variance.

The infinitesimal generator is useful in our context since it can be related to a diffusion process. More precisely, the infinitesimal generator associated with the stochastic diffusion

(15) $$dx_{t}=\mu \left(x_{t}\right)dt+\sigma \left(x_{t}\right)dW_{t}$$

is given by (we refer to Appendix 1 for a derivation by the Itô formula)

(16) $$\left(Gf\right)\left(x\right)=\mu \left(x\right)f^{\prime }\left(x\right)+\frac{1}{2}\sigma^{2}\left(x\right)f^{\prime \prime }\left(x\right).$$

Our strategy is to find a limit of the infinitesimal generator associated with a finite population size process, which corresponds to the form given in Equation 16. We consider a continuous‐time birth–death process with transition rates *T^k^* ^+^ and *T^k^* ^−^ for 0 ≤ *k ≤ N*. Due to the exponentially distributed waiting times, the probability for a single update until time *t* is *λt* exp(−*λt*), where *λ* is the rate of the corresponding exponential clock. Setting *x* = *X*/*N*, the frequency of type‐*A* individuals, we find the infinitesimal generator for the model with finite population size *N*, $G^{N}$, to be of the form

(17) $$\begin{array}{cc} \left(G^{N}f\right)\left(x\right) & =\lim_{\Delta t\to 0}\left\{\frac{1}{\Delta t}\left[\underset{\text{probability}\;\text{of}\;\text{birth}\;\text{of}\;\text{type}\;A\;\text{until}\;\text{time}\;\Delta t}{\underbrace{NT^{xN+}\Delta te^{-NT^{xN+}\Delta t}\left(f\left(x+\frac{1}{N}\right)-f\left(x\right)\right)}}\right.\right.\\ & \left.\;+\left.\underset{\text{probability}\;\text{of}\;\text{death}\;\text{of}\;\text{type}\;A\;\text{until}\;\text{time}\;\Delta t}{\underbrace{NT^{xN-}\Delta te^{-NT^{xN-}\Delta t}\left(f\left(x-\frac{1}{N}\right)-f\left(x\right)\right)}}\right]+\underset{\text{more}\;\text{than}\;\text{one}\;\text{update}\;\text{until}\;\text{time}\;\Delta t}{\underbrace{o(\Delta t)}}\right\}\\ & =N\left(T^{xN+}\left(f\left(x+\frac{1}{N}\right)-f\left(x\right)\right)+T^{xN-}\left(f\left(x-\frac{1}{N}\right)-f\left(x\right)\right)\right). \end{array}$$

We have used the Landau notation *o*(Δ*t*) to summarize processes that scale with order (Δ*t*)^1 +^ *^ε^* for any *ε* > 0. We will also use the Big‐O notation *O*(Δ*t*) for processes that scale with order Δ*t* or higher. Doing a Taylor expansion for large *N* and neglecting the terms of order higher than 1/*N* ^2^, we find

(18) $$\begin{array}{cc} \left(G^{N}f\right)\left(x\right) & =N\left(T^{xN+}\left(f\left(x+\frac{1}{N}\right)‐f\left(x\right)\right)+T^{xN‐}\left(f\left(x‐\frac{1}{N}\right)‐f\left(x\right)\right)\right)\\ & \approx N\left(T^{xN+}\left(f\left(x\right)+\frac{1}{N}f^{\prime }\left(x\right)+\frac{1}{2N^{2}}f^{\prime \prime }\left(x\right)‐f\left(x\right)\right)\right)\\ & \;\left(+T^{xN‐}\left(f\left(x\right)‐\frac{1}{N}f^{\prime }\left(x\right)+\frac{1}{2N^{2}}f^{\prime \prime }\left(x\right)‐f\left(x\right)\right)+O\left(\frac{1}{N^{3}}\right)\right)\\ & =N\left(\left(T^{xN+}‐T^{xN‐}\right)\frac{f^{\prime }\left(x\right)}{N}+\left(T^{xN+}+T^{xN‐}\right)\frac{f^{\prime \prime }\left(x\right)}{2N^{2}}+O\left(\frac{1}{N^{3}}\right)\right)\\ & =\left(T^{xN+}‐T^{xN‐}\right)f^{\prime }\left(x\right)+\frac{1}{2N}\left(T^{xN+}+T^{xN‐}\right)f^{\prime \prime }\left(x\right)+O\left(\frac{1}{N^{2}}\right). \end{array}$$

Translating this equation to a stochastic differential equation, we identify the single components as

(19) $$\mu \left(x\right)=\lim_{N\to \infty }\left(T^{xN+}‐T^{xN‐}\right)\;\text{and}\;\sigma^{2}\left(x\right)=\lim_{N\to \infty }\frac{\left(T^{xN+}+T^{xN‐}\right)}{N}.$$

Note that we have made no assumption on the dependence of the transition probabilities on the frequency *x*, such that this approach is applicable for constant selection, linear frequency dependence arising in two player games (Traulsen et al., 2005) or multiplayer games with polynomial frequency dependence (Gokhale & Traulsen, 2010; Peña et al., 2014).

*Conclusion 2*: For time‐continuous finite population size models with jumps of ±1, that is, a birth–death process, the terms of the continuum limit can be computed by Equation 19.

#### 3.2.1 Example: Moran process with selection and mutation

Returning to our proto‐type processes, we explicitly derive the stochastic differential equation corresponding to the Moran model with selection and mutation. We decouple reproduction and mutation processes, but similar derivations can be made if we assume a coupling of mutations to reproduction events. The selection coefficient is denoted by *s,* and the mutation rates from type *A* to *B* and type *B* to *A* are given by *u_A_*_→_ *_B_* and *u_B_*_→_ *_A_*, respectively. Then, the transition rates are

(20) $$T^{k+}=\left(1+s\right)k\frac{\left(N‐k\right)}{N}+u_{B\to A}\left(N‐k\right),\;\text{and}\;T^{k‐}=\left(N‐k\right)\frac{k}{N}+u_{A\to B}k.$$

Inserting these into Equation 19 yields

(21) $$\begin{array}{c} \mu \left(x\right)=\lim_{N\to \infty }\left(sk\frac{\left(N‐k\right)}{N}+u_{B\to A}\left(N‐k\right)‐u_{A\to B}k\right),\\ \sigma^{2}\left(x\right)=\lim_{N\to \infty }\left(\frac{\left(2+s\right)k\frac{N‐k}{N}+u_{B\to A}\left(N‐k\right)+u_{A\to B}k}{N}\right). \end{array}$$

Depending on the choice of selection and mutation rates, these equations result in different limits. Typically, one is interested in nontrivial limits for these equations, that is, a limit so that not both components equal zero. Often this can be achieved by rescaling time (Section 3.3) and/or defining the strength of selection and mutation in terms of the population size *N*. As an example, we will focus on two specific limits: (a) strong selection and strong mutation and (b) weak selection and weak mutation.

##### Strong selection and mutation

We consider large selection and mutation rates. We assume that *s* and *u_i_* do not depend on *N* but are constant. To obtain a limit equation for the frequency of individuals of type *A*, *x* = *X*/*N*, we rescale time by *N*, that is, *t*→*Nt*, which transforms Equation 21 to

(22) $$\begin{array}{cc} \mu \left(x\right) & =\lim_{N\to \infty }\left(sx\left(1‐x\right)+u_{B\to A}\left(1‐x\right)‐u_{A\to B}x\right)=sx\left(1‐x\right)+u_{B\to A}\left(1‐x\right)‐u_{A\to B}x,\\ \sigma^{2}\left(x\right) & =\lim_{N\to \infty }\left(\frac{2x\left(1‐x\right)+sx\left(1‐x\right)+u_{B\to A}\left(1‐x\right)+u_{A\to B}x}{N}\right)=0. \end{array}$$

The first equation is independent of *N* and the vanishing variance in the second equation implies that the limit process is deterministic. We find the ordinary differential equation

(23) $$dx_{t}=\mu \left(x_{t}\right)dt=sx_{t}\left(1‐x_{t}\right)+u_{B\to A}\left(1‐x_{t}\right)‐u_{A\to B}x_{t},$$

which describes the change in allele frequency in a population under strong selection and mutation over time.

##### Weak selection and mutation

In contrast to the previous scenario, we now assume that both selection and mutation are weak. We let *s* and *u_i_* scale inversely with *N* and define the constants *α* = *sN* and *ν_i_* = *u_i_N*. Inserting these into Equation 21 (and here without rescaling time) yields

(24) $$\begin{array}{c} \mu \left(x\right)=\lim_{N\to \infty }\left(\alpha x\left(1‐x\right)+\nu_{B\to A}\left(1‐x\right)‐\nu_{A\to B}x\right)=\alpha x\left(1‐x\right)+\nu_{B\to A}\left(1‐x\right)‐\nu_{A\to B}x,\\ \sigma^{2}\left(x\right)=\lim_{N\to \infty }\left(2x\left(1‐x\right)+\frac{\alpha x\left(1‐x\right)+\nu_{A\to B}\left(1‐x\right)+\nu_{A\to B}x}{N}\right)=2x\left(1‐x\right), \end{array}$$

which gives the diffusion limit

(25) $$dx_{t}=\left(\alpha x_{t}\left(1‐x_{t}\right)+\nu_{B\to A}\left(1‐x_{t}\right)‐\nu_{A\to B}x_{t}\right)dt+\sqrt{2x_{t}\left(1‐x_{t}\right)}dW_{t}.$$

Note that compared to the Wright–Fisher diffusion in Equation 13 this limit has twice as much variance. This difference is explained by the different sampling schemes in the individual‐based description of the model. To see this, we assume no selection, *s* = 0, and no mutation, *u_A_*_→_ *_B_* = *u_B_*_→_ *_A_* = 0.

In the Wright–Fisher process, individuals are updated by binomial sampling. The variance of this sampling procedure is *Nx*(1 − *x*), where the factor *N* vanishes by rescaling the time. This gives *σ* ^2^ (*x*) = *x*(1 − *x*).

In the Moran model, or more generally for a birth–death process, the variance is computed by the sum of the transition rates, cf. Equation 19. In our example, both transitions happen at rate 1, which explains the additional factor 2. The difference between the variances is therefore a result of the different sampling schemes of the individual‐based models.

As a consequence of this difference in the variance *σ* ^2^ (*x*) between the two models, the Moran diffusion limit progresses twice as fast as the Wright–Fisher diffusion limit which can be seen by the scaling property of the Brownian motion. In terms of the original discrete processes, this means that *N* individual jumps, like in the Moran process, accumulate more variance than one update of the whole population, like in the Wright–Fisher process. The sampling therefore determines the variance and consequently the speed of the continuum limit.

*Conclusion 3*: The Moran process, by definition, has the same mean behavior as the Wright–Fisher model. However, its variance in the diffusion limit is twice the variance of the corresponding Wright–Fisher diffusion. This difference arises from the different sampling schemes of the individual‐based models.

### 3.3 Change of time scales in the derivation of a continuum limit

In the derivation of the continuum limit, we have repeatedly rescaled time to obtain a nontrivial limit, for example, right before Equations (13), (22) and 22. Rescaling the time speeds up (or slows down) the original process so that the dynamics of interest, for example, allele frequency changes, become observable. For example, if the dynamics were to be very fast in the original process, we would need to slow down time appropriately to observe the changes of the quantity of interest more gradually. In general, we are free to chose any scaling of time. However, it is important to keep in mind the scaling when interpreting results obtained in the limiting process in terms of the original process. Especially so, if one is interested in quantities involving time, for example, fixation or extinction times.

*Conclusion 4*: Different assumptions on the model dynamics, for example, on selection and mutation, can lead to different continuum limits on the population level. To identify parameter combinations that result in a reasonable continuum limit, one needs to study Equation 19 to match the orders of the scaling parameter. Rescaling time gives an additional degree of freedom when trying to match these orders to obtain a reasonable limit.

## 4 DIFFUSION APPROXIMATION

We have seen that if we let the population size *N* tend to infinity, we can derive a (stochastic) differential equation describing the studied evolutionary or ecological process. A natural question that arises is how these results relate to finite population size models. To study this difference between the finite population process and the continuum limit, we consider the logistic growth equation. The transition rates are given by

(26) $$T^{j+}=\beta j\;\text{and}\;T^{j‐}=j\left(\delta +\frac{\gamma (j‐1)}{K}\right).$$

Repeating the steps from the previous section with *y* = *j*/*K*, we find the following expressions for the infinitesimal mean and variance:

(27) $$\begin{array}{c} \mu \left(y\right)=\lim_{K\to \infty }\left(\frac{T^{\mathrm{yK}+}‐T^{\mathrm{yK}‐}}{K}\right)=\left(\beta ‐\delta \right)y\left(1‐\frac{\gamma y}{\left(\beta ‐\delta \right)}\right),\\ \sigma^{2}\left(y\right)=\lim_{K\to \infty }\left(\frac{T^{\mathrm{yK}+}+T^{\mathrm{yK}‐}}{K^{2}}\right)=\lim_{K\to \infty }\frac{(\beta +\delta +\gamma y)y}{K}=0. \end{array}$$

Thus, the classical deterministic logistic equation is obtained in the infinite population size limit, *K* → ∞:

(28) $$dy_{t}=\left(\beta ‐\delta \right)y_{t}\left(1‐\frac{\gamma y_{t}}{\beta ‐\delta }\right)dt=\mu \left(y_{t}\right)dt.$$

How well does the finite population size description approximate this deterministic limit? One way to approach this question is to simply not take the limit of *K* to infinity. The finite population size logistic equation, derived from Equation 27, is then approximated by

(29) $$dy_{t}^{K}=\mu \left(y_{t}^{K}\right)dt+\sqrt{\frac{\left(\beta +\delta +\gamma y_{t}^{K}\right)y_{t}^{K}}{K}}dW_{t},$$

where the superscript *K* in $y_{t}^{K}$ indicates the order of magnitude of the carrying capacity.

The approximation in Equation 29 is called *Diffusion approximation*. One can prove formally that this approximation, under the assumptions that the function *μ*(*x*) and *σ* ^2^(*x*) are (twice) continuously differentiable, performs equally well as a more accurate analysis based on the central limit theorem (Ethier & Kurtz, 1986, Theorem 11.3.2). For a rigorous discussion of diffusion approximations and their relation to the central limit theorem, we refer to Ethier and Kurtz (1986, Chapter 11).

**FIGURE 1:**
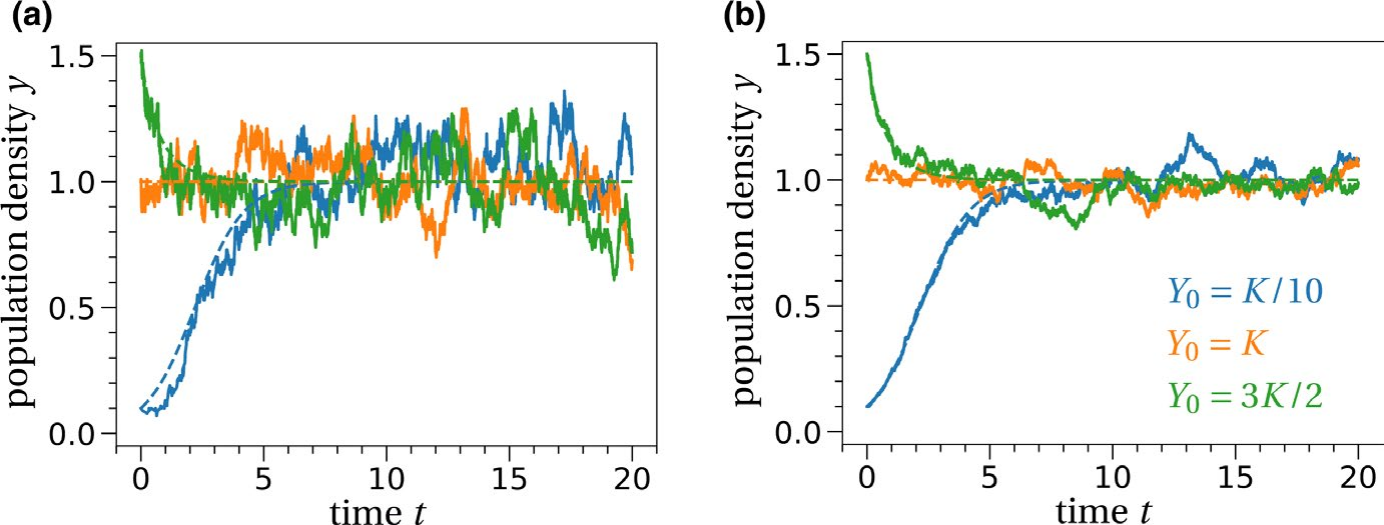
Individual‐based simulations of the logistic growth model. (a) For low population sizes, the individual‐based simulation (solid lines) fluctuates strongly around the deterministic solution of the population (dashed lines) given by Equation 28. (b) Increasing the scaling parameter *K*, the stochastic fluctuations around the deterministic prediction decrease, until eventually the individual‐based simulation is indistinguishable from the deterministic curve. The parameter values are chosen as follows: *β* = 2, *δ* = 1, *γ* = 1, and (a) K=100, (b) K=1,000. The initial population sizes are stated in subfigure (b)

In terms of performance of the diffusion approximation, the population size measure *K* does not need to be very large for the individual‐based model to approach the deterministic limit (*K* ≈ 1,000 is enough in this example, Figure 1).

*Conclusion 5*: Not taking the limit in Equation 19 yields the diffusion approximation of the studied model. This approximation is a stochastic differential equation (Equation 29) where the variance (typically) scales inversely with the square root of the scaling parameter.

## 5 STATIONARY DISTRIBUTIONS

**FIGURE 2:**
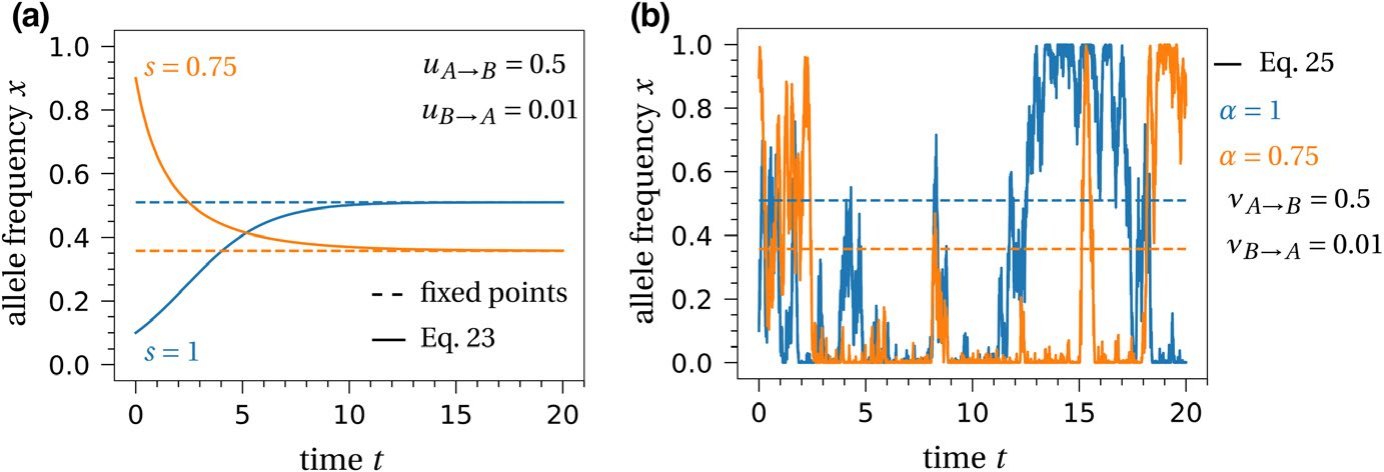
Allele frequency dynamics with selection and mutation. (a) The deterministic system given by Equation 23 converges to the fixed point (dashed line) and remains there. (b) The stochastic process given by Equation 25 fluctuates strongly in frequency space and for the chosen parameter values spends most time close to the monotypic states *x* = 0 and *x* = 1

For the Moran model, we have derived two different limits that differ in their assumptions on selection and mutation. If both selection and mutation are strong, the infinite population size limit is an ordinary differential equation. For weak selection and weak mutation, we derived a stochastic differential equation. One qualitative difference between these two limits is that trajectories of the deterministic limit will always converge to a fixed point (other limits are possible in general, e.g., limit cycles) while the stochastic differential equation fluctuates indefinitely for positive mutation rates. The deterministic fixed point of the Moran model is given by the solution of Equation 23 equal to zero. In our example, a single fixed point x* lies within the interval between 0 and 1 and is stable. Therefore, all trajectories will approach this value, for example, Figure 2a.

In contrast, Equation 25 is a stochastic equation. Thus, even if the trajectories approach or hit the deterministic fixed point they will not stay there due to the stochasticity of the Brownian motion, cf. Figure 2b. Still, we can make predictions about the time a trajectory spends in certain allele configurations. This information is summarized in the stationary distribution, the stochastic equivalent of a deterministic fixed point. If the initial state of the population is given by the stationary distribution, then the distribution of all future time points will not change. For birth–death processes, the stationary distribution can be calculated based on detailed balance, that is, the incoming and outgoing rates need to be equal for every state of the process (Antal et al., 2009; Claussen & Traulsen, 2005; Gardiner, 2004). Formally, the stationary distribution, denoted *ψ*, is defined as the solution of

(30) $$\frac{d}{dt}E[f(x_{t})|x_{0}∼\psi ]=0,$$

where *x*_0_∼*ψ* denotes that *x*_0_ is distributed according to *ψ* and *f* is an arbitrary function. Importantly, this condition means that the distribution of allele frequencies does not change over time because its derivative in time is zero (for any choice of *f*). The above equation can be solved with the infinitesimal generator (Etheridge (2012, Chapter 3.6)). The solution is expressed in terms of the speed measure density *m*(*x*), which we introduce in Box 1, and is given by

(31) $$\psi (x)=\frac{m(x)}{\begin{array}{c} \int_{0}^{1} \end{array}m(y)dy}.$$

Intuitively, the speed measure at a point *x*, *m*(*x*), quantifies the time which the process spends in this state. Therefore, *ψ*(*x*) is nothing but the average time spent in state *x*.

*Conclusion 6*: The stationary distribution of a one‐dimensional diffusion can be expressed in terms of the density of the speed measure m(x) through Equation 31. The density of the speed measure is given by the scale function corresponding to the stochastic diffusion process, Equations (32), (34) and (22), (34) in Box 1.

BOX 1 **Scale function and speed measure of a one‐dimensional diffusion**

A one‐dimensional stochastic diffusion can be transformed into a standard Brownian motion. Since the Brownian motion is well‐studied, a lot of results can then be translated to the stochastic diffusion by the transformation functions, the *scale function,* and the *speed measure*.

First, we rescale the space of the original process by the scale function. It is defined by

(32) $$S(x)=\begin{array}{c} \int_{x}^{} \end{array}\exp\left(‐2\begin{array}{c} \int_{y}^{} \end{array}\frac{\mu (z)}{\sigma^{2}(z)}dz\right)dy,\;x\in \left(0,\;1\right),$$

where the lower boundaries of the integrals can be chosen arbitrarily. The name of this function derives from the fact that for a one‐dimensional diffusion *x_t_* satisfying

(33) $$dx_{t}=\mu \left(x_{t}\right)dt+\sigma \left(x_{t}\right)dW_{t},$$

the scaled process *S*(*x_t_*) becomes a time‐changed Brownian motion on the interval [*S*(0), *S*(1)], that is, there is no deterministic contribution in the scaled process. The process *S*(*x_t_*) is a Brownian motion with a “non‐standard” time scale. To map this time‐changed Brownian motion to the time scale of a standard Brownian motion, one needs to rescale time by the speed measure *M*. It defines how much faster (or slower) the process *S*(*x_t_*) is evolving compared to a standard Brownian motion. The speed measure is defined by

(34) $$M(x)=\begin{array}{c} \int_{}^{x} \end{array}m(y)dy,\;\text{with}\;m(y)=\frac{1}{\sigma^{2}(y)S^{\prime }(y)}$$

the density of the speed measure. The time is then rescaled by $\tau (t)=\int_{0}^{t}\;m(S(x_{s}))ds.$

Compactly written, we have changed the stochastic diffusion *x_t_* to the standard Brownian motion by the following steps:

$$\begin{array}{c} x_{t}\\ (\text{stochastic}\\ \text{diffusion}) \end{array}\overset{x\mapsto S(x)}{⟶}\begin{array}{c} S(x_{t})=B_{t}\\ (\text{time - changed}\\ \text{Brownian}\;\text{motion}) \end{array}\overset{t\mapsto \tau (t)}{⟶}\begin{array}{c} B_{\tau (t)}\\ (\text{standard}\\ \text{Brownian}\;\text{motion}) \end{array}.$$

### 5.1 Stationary distribution of the Wright–Fisher diffusion

As an example, let us consider the Wright–Fisher diffusion with selection and mutation as derived in Equation 13 (and Equation 25 when derived from the Moran model), that is,

(35) $$dx_{t}=\left(\alpha x_{t}\left(1‐x_{t}\right)+\nu_{B\to A}\left(1‐x_{t}\right)‐\nu_{A\to B}x_{t}\right)dt+\sqrt{x_{t}\left(1‐x_{t}\right)}dW_{t}.$$

Computing Equation 31 with help of the quantities defined in Box 1, one obtains

(36) $$\psi \left(x\right)=e^{2\alpha x}x^{2\nu_{B\to A}‐1}{\left(1‐x\right)}^{2\nu_{A\to B}‐1}\frac{\Gamma (2\left(\nu_{A\to B}+\nu_{B\to A}\right))}{\Gamma \left(2\nu_{A\to B}\right)\Gamma {\left(2\nu_{B\to A}\right)}_{1}F_{1}\left(2\nu_{B\to A},2\left(\nu_{A\to B}+\nu_{B\to A}\right),\alpha \right)},$$

where Γ(x) is the Gamma function and _1_ *F*_1_ (*a*, *b*, *z*) is the generalized hypergeometric function.

**FIGURE 3:**
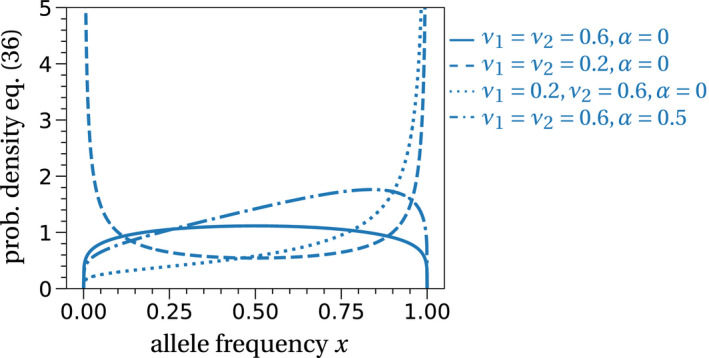
Stationary distribution of the Wright–Fisher diffusion with selection and mutation. The lines are given by Equation 36. Larger mutation rates accumulate more probability on intermediate allele frequencies (compare solid and dashed lines). Selection (or asymmetric mutation) skews the stationary distribution toward the selectively favored type (or type with the lower mutation rate), dash‐dotted (dotted) line

The equation itself does not provide much insight. To illustrate the possible shapes of stationary distributions, we plot several choices of mutation rates and selection coefficients in Figure 3. We see that for higher mutation rates, more probability mass is allocated to intermediate allele frequencies (compare the solid and dashed lines). In this case, the Wright–Fisher diffusion spends more time in states of coexistence than in monotypic states (the boundaries of the allele frequency space in Figure 3) because temporary extinction events are prevented by recurrent mutations. If the mutation rates are asymmetric (dotted line), the stationary distribution is skewed toward the type with the lower mutation rate. If one type is favored selectively (dash‐dotted line), the stationary distribution is skewed toward the favored type.

Stationary distributions are the stochastic equivalents of deterministic fixed points and as such provide a basic description and a starting point for further analysis of the qualitative behavior of a stochastic model, especially in situations where polymorphisms of alleles, coexistence of species, or spatial population distributions are to be expected (e.g., Czuppon & Rogers, 2019; Gaston & He, 2002; Lehmann, 2012; Polansky, 1979; Turelli, 1981).

### 5.2 Quasi‐stationary distribution of the logistic process

Next, we consider the diffusion approximation of the logistic growth model, that is,

(37) $$dy_{t}^{K}=\left(\beta ‐\delta ‐\gamma y_{t}^{K}\right)y_{t}^{K}dt+\sqrt{\frac{\left(\beta +\delta +\gamma y_{t}^{K}\right)y_{t}^{K}}{K}}dW_{t}.$$

The logistic process for finite *K* has a (unique) absorbing state, *y* = 0 because there is no transition from this state to positive population densities. Once the population went extinct, it remains so. Since the extinction state is accessible from all values *y* > 0 (and because the population size remains finite for all times), the population will go extinct with probability 1. The only stationary distribution is the point measure on 0, that is, *ψ* ∼ *δ*_0_ (*δ_x_* is the Dirac measure at point *x*).

In contrast, the positive deterministic population equilibrium, y* = (*β* − *δ*)/*γ*, is a stable fixed point of the deterministic system. Considering large values of the deterministic equilibrium (K≫1), we expect the finite population size process from Equation 37 to remain close to this value for long times. In fact, the expected extinction time of the logistic growth process when started in the positive population equilibrium is of order exp(*K*) (Champagnat, 2006). This suggests that the process will be in a quasi‐stationary state, that is, before its extinction the population is described by the stationary distribution of the corresponding logistic process conditioned on nonextinction.

Formally, the quasi‐stationary distribution is computed by conditioning the original process on its survival. This means that the transition rates change and the novel process can be analyzed by the techniques described above. However, this method goes beyond the scope of this manuscript. For a theoretical treatment of this topic in the context of the logistic equation, we refer to Cattiaux et al. (2009), Assaf et al. (2010), Méléard and Villemonais (2012). For a general review on methods related to quasi‐stationary distributions, see Ovaskainen and Meerson (2010).

Another way to approximate the quasi‐stationary distribution when extinction is very unlikely for long times (which is the case for large *K*) is provided by the central limit theorem (sometimes also called linear noise approximation in this context). Here, the distribution of the process is derived from its local dynamics around the deterministic fixed point y* (Ethier & Kurtz, 1986; van Kampen, 2007). The underlying assumption is that the population stays close to its positive steady state and just slightly fluctuates around this value. This is only a valid assumption when the probability of extinction within the studied time frame is essentially zero. These small fluctuations are described by a Gaussian distribution. Formally this translates to

(38) $$y_{t}^{K}\approx y_{t}+\frac{1}{\sqrt{K}}U,$$

where *U* is a Gaussian random variable and *y_t_* the deterministic trajectory. Writing *μ*(*y*) = (*β* − *δ* − *γy*)*y* and *σ* ^2^ (*y*) = (*β* + *δ* + *γy*)*y*, the dynamics of *U* can be rewritten as

(39) $$\begin{array}{cc} \frac{1}{\sqrt{K}}dU & \approx dy_{t}^{K}‐dy_{t}=\left(\mu \left(y_{t}^{K}\right)‐\mu \left(y_{t}\right)\right)dt+\sqrt{\frac{\sigma^{2}\left(y_{t}^{K}\right)}{K}}dW_{t}\\ & \approx \mu^{\prime }\left(y_{t}\right)\left(y_{t}^{K}‐y_{t}\right)dt+\sqrt{\frac{\sigma^{2}\left(y_{t}^{K}\right)}{K}}dW_{t}\;\left(\text{Taylor}\;\text{series}\;\text{approximation}\right)\\ & \approx \frac{1}{\sqrt{K}}\left(\mu^{\prime }\left(y_{t}\right)Udt+\sigma \left(y_{t}^{K}\right)dW_{t}\right). \end{array}$$

Evaluating the process *U* at $y_{t}^{K}=y_{t}=y^{*}$, we obtain a description of the variance in the deterministic fixed point. For this fixed choice of *y_t_* and $y_{t}^{K}$, *U* becomes an Ornstein–Uhlenbeck process. Its stationary distribution is then given by

(40) $$\psi^{U}∼N\left(0,‐\frac{\sigma^{2}(y^{*})}{2\mu^{\prime }(y^{*})}\right).$$

This distribution describes the fluctuations of the process $y_{t}^{K}$ around the deterministic steady state y*. Therefore, when plugging the distribution *ψ^U^* into the original process from Equation 38, we find the quasi‐stationary distribution of $y_{t}^{K}$ around the deterministic equilibrium y* which yields

(41) $$\psi ∼N\left(y^{*},‐\frac{\sigma^{2}(y^{*})}{2K\mu^{\prime }(y^{*})}\right).$$

For increasing population sizes *K*, the variance is decreasing and vanishes in the limit *K*→∞, as is to be expected by the deterministic limit.

*Conclusion 7*: If the deterministic process has a stable steady state but is almost surely going extinct for finite population sizes, a quasi‐stationary distribution can be computed to describe the behavior of the process conditioned on survival. If the extinction probability is very low, an approximation of this distribution is given by the linear noise approximation where the variance around the deterministic steady state is modeled by the Ornstein–Uhlenbeck process derived from Equation 39.

## 6 FIXATION PROBABILITIES

We have seen that stochastic descriptions of processes can lead to outcomes that are different from their deterministic counterparts. Here, we study one of these phenomena: the probability for a certain type to become fixed in a population. For one‐dimensional stochastic differential equations, fixation probabilities can be computed explicitly. As before, we denote by *x_t_* the frequency of type *A* individuals at time *t* ≥ 0 in the population. We use the fact that the mean of the scaled process *S*(*x_t_*) in Equation 32 does not change over time. Stochastic processes with this property are called *martingales*. With $p_{\text{fix}}\left(x_{0}\right)=P\left(x_{\infty }=1|x_{0}\right)$ we find

(42) $$\begin{array}{c} S(x_{0})=E[S(x_{t})|x_{0}]\overset{t\gg 1}{=}p_{\text{fix}}(x_{0})S(1)+(1‐p_{\mathrm{fix}}(x_{0}))S(0)\\ \Leftrightarrow \;p_{\text{fix}}(x_{0})=\frac{S(x_{0})‐S(0)}{S(1)‐S(0)}, \end{array}$$

where we have used the martingale property of the scaled process *S*(*x*) in the first equality. The second equality is explained by the process being absorbed at one of the two boundaries *x* = 0 or *x* = 1 at large times. For a formal derivation, we refer to Otto and Day (2007, Chapter 15.3.3) or Etheridge (2012, Lemma 3.14).

As an example, let us consider the Wright–Fisher diffusion with selection and without mutations (*ν_A_*_→_ *_B_* = *ν_B_*_→_ *_A_* = 0) given in Equation 35. We have *μ*(*x*) = α*x*(1 − *x*) and *σ* ^2^(*x*) = *x*(1 − *x*) such that the scale function simplifies to

(43) $$S(x)=\begin{array}{c} \int_{}^{x} \end{array}\exp\left(‐2\begin{array}{c} \int_{}^{y} \end{array}\frac{\alpha z(1‐z)}{z(1‐z)}dz\right)dy=‐\frac{1}{2\alpha }\exp(‐2\alpha x).$$

Recalling the definition of *α* = *sN* for a finite population of size *N* and plugging this into Equation 42 yields

(44) $$\begin{array}{cc} P_{x_{0}}(x_{\infty }=1) & =\frac{1‐e^{‐2\alpha x_{0}}}{1‐e^{‐2\alpha }}=\frac{1‐e^{‐2sx_{0}N}}{1‐e^{‐2sN}}\\ & \overset{Ns⪢1}{\approx }\;1‐e^{‐2sx_{0}N}\\ & \overset{s⪡1}{\approx }\;2sx_{0}N, \end{array}$$

which for *x*_0_ = 1/*N* becomes $P_{1/N}\left(x_{\infty }=1\right)\approx 2s$, the result of Haldane for the fixation of a single mutant copy in a population of size *N* (Haldane, 1927). The first line of Equation 44, the classical result of fixation probabilities when derived from diffusion theory, and its applicability has been the subject of extensive research, for example, Bürger and Ewens (1995) and references therein; for a more general review on fixation probabilities, we refer to Patwa and Wahl (2008).

Of course, the fixation probability can also be calculated for more complicated stochastic differential equations where the sign of the deterministic dynamics *μ*(*x*) depends on the population configuration. Most classically, these frequency‐dependent problems were studied in deterministic evolutionary game theory introduced by Maynard Smith and Price (1973) (see also Hofbauer and Sigmund (1998) for an introduction to evolutionary game dynamics). In Appendix 3, we (re‐)derive the fixation probability in case of frequency‐dependent selection.

*Conclusion 8*: The fixation probability of a one‐dimensional diffusion is given by the scale function as stated in Equation 42.

## 7 MEAN TIME TO FIXATION

A related quantity of interest is the expected time to fixation (or extinction from the other type's point of view), that is, the average time of coexistence of two types. Again, the calculation relies on a special function, this time *Green's function G*(*x*, *y*), which can be interpreted as the average time that a diffusion started in *x* spends in the interval [*y*, *y* + *dy*) before reaching one of the boundaries (Etheridge, 2012, Chapter 3.5). It is therefore also called sojourn time density (Ewens, 2004). It is defined as

(45) $$G\left(x,y\right)=\left\{\begin{array}{c} 2\frac{S(x)‐S(0)}{S(1)‐S(0)}\left(S\left(1\right)‐S\left(y\right)\right)m\left(y\right),\;0\leq x\leq y\leq 1,\\ 2\frac{S(1)‐S(x)}{S(1)‐S(0)}\left(S\left(y\right)‐S\left(0\right)\right)m\left(y\right),\;0\leq y\leq x\leq 1, \end{array}\right)$$

where *S*(*x*) is the previously defined scale function and *m*(*x*) denotes the speed measure density (Box 1).

The expected time to fixation for a process started at frequency *x*, denoted $E_{x}\left[\tau \right]$, is then given by (Ewens, 2004, section 4.4).

(46) $$E_{x}\left[\tau \right]=\begin{array}{c} \int_{0}^{1} \end{array}G(x,y)dy.$$

This integral corresponds to the summation of the sojourn times in the discrete case, for example, we refer to Ohtsuki et al. (2007) for an application in finite populations. In some cases, the result of this equation yields an analytically tractable result, for example, for the neutral Wright–Fisher diffusion

(47) $$dx_{t}=\sqrt{x_{t}\left(1‐x_{t}\right)}dW_{t}.$$

In this case, the scale function and speed measure density are given by

(48) $$S\left(x\right)=x\;\text{and}\;m\left(x\right)=\frac{1}{x(1‐x)}.$$

Then, the expected time to fixation of one of the two alleles can be expressed as

(49) $$\begin{array}{cc} E_{x}\left[\tau \right] & =\int_{0}^{x}2\left(1‐x\right)\frac{y}{y(1‐y)}dy+\int_{x}^{1}2x\frac{\left(1‐y\right)}{y(1‐y)}dy\\ & =2\left(1‐x\right)\ln\left({\left(1‐x\right)}^{‐1}\right)+2x\ln\left(x^{‐1}\right). \end{array}$$

In Appendix 3, we consider the more involved example of frequency‐dependent selection (Altrock & Traulsen, 2009; Pfaffelhuber & Wakolbinger, 2018).

Similarly to fixation probabilities, the mean time to fixation has been studied extensively through stochastic diffusions, for example, Kimura and Ohta (1969). It is especially important in population genetics where one is interested in the time to extinction or fixation of newly arising alleles (van Herwaarden & van der Wal, 2002). In ecology, the mean time to extinction or fixation is for example applied in the context of population extinction (Lande, 1994) and speciation events (Yamaguchi & Iwasa, 2013).

*Conclusion 9*: Expected unconditional fixation times, that is, the expected time of coexistence of two types in a population, can be calculated by integrating over Green's function (the mean occupation time of a certain frequency until extinction), as shown in Equation 46.

## 8 DISCUSSION AND CONCLUSION

We have outlined how to derive a stochastic differential equation from an individual‐based description of two classical models in evolutionary theory and theoretical ecology, the Wright–Fisher diffusion and the logistic growth equation. The resulting stochastic differential equations in one dimension describe the dynamics of the allele frequency and population density, respectively. Using probabilistic properties of this equation, that is, transforming it to a standard Brownian motion (Box 1), it is possible to analytically derive the (quasi‐) stationary distribution, fixation probability, and the mean time to fixation. As an example, we derived these quantities for the Wright–Fisher diffusion.

The diffusion process emerges as the infinite population size limit. However, as we have shown in Section 4, one can also derive a finite population size approximation of the dynamics, the diffusion approximation. The fixation probability, mean extinction time, and stationary distribution are accessible by the same means as for the continuum limit. Applications of diffusion approximations are abundant and cover diverse topics (e.g., Assaf & Mobilia, 2011; Constable et al., 2016; Czuppon & Gokhale, 2018; Czuppon & Traulsen, 2018; Débarre & Otto, 2016; Houchmandzadeh, 2015; Kang & Park, 2017; Koopmann et al., 2017; McLeod & Day, 2019; Parsons et al., 2018; Reichenbach et al., 2007; Schenk et al., 2020; Serrao & Täuber, 2017; Traulsen et al., 2005).

Apart from the fixation probability and the mean time to fixation, the (quasi‐)stationary distribution is a commonly used measure to describe stochastic processes. Its calculation through the speed measure of the associated scaled process (Box 1) is (in many cases) numerically straightforward. If the process has an absorbing state, for example, an extinction boundary of the population, the stationary distribution is not meaningful. Here, the quasi‐stationary distribution describes the stationary distribution conditioned on the survival of the population. For negligible extinction probabilities, that is, very large survival probabilities of the population, the functional central limit theorem (or linear noise approximation) can be used to approximate this quasi‐stationary distribution. In the theoretical biology literature, this method is frequently used in models of gene regulatory networks (see Anderson and Kurtz (2015) for a mathematical introduction), and less so in the context of ecology or evolution (e.g., Boettiger et al. (2010); Kopp et al. (2018); Wienand et al. (2018); Czuppon and Constable (2019); and Assaf and Meerson (2017) for a review of the physics literature related to this topic).

Lastly, we did not cover multi‐dimensional or spatially explicit stochastic differential equations in this methods review. These processes are often much more complicated to analyze. Here, we aimed to give a brief introduction into the derivation of a continuum limit from an individual‐based model. We hope, that with our basic comparisons between different approaches used in different subfields of theoretical biology, we help to clarify the common foundation, the individual‐based model, on which these different methods are based.

## CONFLICT OF INTEREST

The authors declare no conflict of interest.

## AUTHOR CONTRIBUTION

**Peter Czuppon:** Conceptualization (equal); methodology (lead); writing–original draft (lead); writing–review and editing (equal). **Arne Traulsen:** Conceptualization (equal); writing–review and editing (equal).

## Data Availability

No data are used.

## APPENDIX 1

### INFINITESIMAL MEAN AND VARIANCE

Given the stochastic differential equation

(50) $$dx_{t}=\mu \left(x_{t}\right)dt+\sigma \left(x_{t}\right)dW_{t},$$

the corresponding infinitesimal generator is defined by

(51) $$\left(Gf\right)\left(x\right)=\mu \left(x\right)f^{\prime }\left(x\right)+\frac{1}{2}\sigma^{2}\left(x\right)f^{\prime \prime }\left(x\right).$$

The connection between the infinitesimal generator and its associated stochastic differential equation is outlined in more detail in, for example, Kallenberg (2002, Chapter 23). Briefly, one needs to apply Itô's formula (Kallenberg, 2002, Theorem 17.18) to the process *f*(*x_t_*), where *x_t_* solves the stochastic differential equation in Equation 50:

(52) $$df(x_{t})\;\overset{\mathrm{It}\hat{o}}{=}\;f^{\prime }(x_{t})\;dx_{t}+\frac{\sigma^{2}(x_{t})}{2}f^{\prime \prime }(x_{t})\;dt\;\overset{\mathrm{Eq}.(50)}{=}\;\left(\mu (x_{t})f^{\prime }(x_{t})+\frac{\sigma^{2}(x_{t})}{2}f^{\prime \prime }(x_{t})\right)dt+\sigma (x_{t})\;dW_{t}.$$

Taking the expectation yields the result since the last term on the right‐hand side vanishes (the mean of a standard Brownian motion is zero).

Additionally, we show that *μ*(*x*) is indeed the infinitesimal mean of the stochastic process with infinitesimal generator G as given in Equation 51. Setting *f*_1_(*x*) = *x* (and thus $f_{1}^{\prime \prime }\;\left(x\right)=0$) we find for the infinitesimal change of the mean

(53) $$\lim_{\Delta t\to 0}\frac{1}{\Delta t}E[x_{\Delta t}‐x_{0}|x_{0}=x]\;\overset{\text{def}.}{=}\;(Gf_{1})(x)\;\overset{\text{Eq}.(51)}{=}\;\mu (x).$$

Similarly, we see that *σ* ^2^(*x*) is the infinitesimal variance. With *f*_2_(*x*) = *x* ^2^, we have

(54) $$\begin{array}{cc} \underset{\Delta t\to 0}{\text{lim}}\frac{1}{\Delta t}V[(x_{\Delta t}‐x_{0})|x_{0}=x] & \overset{\text{def}.}{=}\underset{\Delta t\to 0}{\text{lim}}\frac{1}{\Delta t}E[(x_{\Delta t}‐x_{0}‐E[x_{\Delta t}‐x_{0}|x_{0}=x]){\;}^{2}|x_{0}=x]\\ & =\underset{\Delta t\to 0}{\text{lim}}\frac{1}{\Delta t}\left(E[(x_{\Delta t}‐x_{0}){\;}^{2}|x_{0}=x]‐\underset{=O((\Delta t){\;}^{2})}{\underbrace{(E[x_{\Delta t}‐x_{0}|x_{0}=x]){\;}^{2}}}\right)\\ & =\lim_{\Delta t\to 0}\frac{1}{\Delta t}E[x_{\Delta t}^{2}‐x_{0}^{2}‐2x_{0}(x_{\Delta t}‐x_{0})|x_{0}=x]\\ & \overset{\text{def}.}{=}(Gf_{2})(x)‐2x(Gf_{1})(x)\\ & \overset{\text{Eq}.(51)}{=}2x\mu (x)+\sigma^{2}(x)‐2x\mu (x)=\sigma^{2}(x). \end{array}$$

This justifies that calculating the infinitesimal mean and variance (right‐hand sides in Equation 9) indeed yields the functions *μ* and *σ* ^2^ of the diffusion.

## APPENDIX 2

### DERIVING A STOCHASTIC DIFFERENTIAL EQUATION FROM THE WRIGHT–FISHER MODEL WITH SELECTION AND MUTATION

In the main text, we have derived the Wright–Fisher diffusion in the absence of selection and mutation. Here, we provide the calculation steps when including both these processes.

We say that type *A* alleles are beneficial (deleterious) if *s* > 0 (*s* < 0). Given that there are *k* type *A* individuals in the population, the probability for an offspring to choose a type *A* individual as a parent is given by

(55) $$p_{k}=\frac{(1+s)k}{(1+s)k+N‐k}.$$

We can also add mutations to the Wright–Fisher model, that is, type *A* individuals can mutate to type *B* and vice versa. We set *u_A_*_→_ *_B_* as the probability to mutate from type *A* to *B* and *u_B_*_→_ *_A_* as the mutation probability from *B* to *A*. Then, the probability for an individual to be of type *A* given *k* type *A* individuals in the parental generation reads

(56) $$p_{k}=\frac{\left(1+s\right)k\left(1‐u_{A\to B}\right)}{(1+s)k+N‐k}+\frac{u_{B\to A}\left(N‐k\right)}{(1+s)k+N‐k}.$$

In this model, mutation is intimately connected with the reproduction mechanism. For the Moran model, compare Section 3, these processes do not necessarily need to be coupled (even though this would, biologically speaking, make the most sense).

Following the same methodology as for the neutral Wright–Fisher model in the main text, we can derive a diffusion process by computing the infinitesimal mean and variance. Writing *x_t_* = *X_t_*/*N* and setting Δ*t* = 1/*N*, we obtain for the infinitesimal change in allele frequency

(57) $$\begin{array}{cc} \frac{1}{\Delta t}E\left[x_{\Delta t}‐x_{0}|x_{0}=x\right] & =E\left[X_{\Delta t}‐X_{0}|X_{0}=k\right]=\left(Np_{k}‐k\right)\\ & =\left(N\frac{\left(1+s\right)k\left(1‐u_{A\to B}\right)}{N+sk}+N\frac{u_{B\to A}\left(N‐k\right)}{N+sk}‐k\right). \end{array}$$

This is a rather unhandy expression. However, we can make further progress by assuming that selection and mutation are weak, that is, we set *s* = *α*/*N* and *u_i_* = *ν_i_*/*N*. Rewriting the equation in terms of *α* and *ν_i_*, expanding the equation in terms of 1/*N*, and neglecting terms of order 1/*N* ^3^ and higher we find

(58) $$\begin{array}{cc} \frac{1}{\Delta t}E\left[x_{\Delta t}‐x_{0}|x_{0}=x\right] & =N\frac{\left(1+\frac{\alpha }{N}\right)k\left(1‐\frac{\nu_{A\to B}}{N}\right)}{N+\frac{k\alpha }{N}}+N\frac{\frac{\nu_{B\to A}}{N}\left(N‐k\right)}{N+\frac{k\alpha }{N}}‐k\\ & =k+\frac{\alpha k}{N}‐\frac{\nu_{A\to B}k}{N}+\frac{\nu_{B\to A}\left(N‐k\right)}{N}‐k‐\alpha \frac{k^{2}}{N^{2}}+O\left(\frac{1}{N^{3}}\right)\\ & =\alpha x\left(1‐x\right)‐\nu_{A\to B}x+\nu_{B\to A}\left(1‐x\right)+O\left(\frac{1}{N^{3}}\right). \end{array}$$

Thus, for the infinitesimal mean in the infinite population size limit, we find

(59) $$\mu \left(x\right)=\alpha x\left(1‐x\right)‐\nu_{A\to B}x+\nu_{B\to A}\left(1‐x\right).$$

The infinitesimal variance in terms of *α* and *ν_i_* derives to

(60) $$\begin{array}{cc} \frac{1}{\Delta t}E\left[{\left(\Delta x_{t}\right)}^{2}|x_{0}=x\right] & =\frac{1}{N}\left(E\left[X_{t+\frac{1}{N}}^{2}‐2X_{t}X_{t+\frac{1}{N}}+X_{t}^{2}|X_{0}=k\right]\right)\\ & =\frac{1}{N}\left(V\left[X_{t+\frac{1}{N}}|X_{0}=k\right]+{\left(E\left[X_{t+\frac{1}{N}}|X_{0}=k\right]‐k\right)}^{2}\right)\\ & =p_{k}\left(1‐p_{k}\right)+\frac{1}{N}\mu^{2}\left(x\right)\\ & =x\left(1‐x\right)+O\left(\frac{1}{N}\right), \end{array}$$

where we used

(61) $$p_{k}=\frac{\left(k+\frac{k\alpha }{N}‐\frac{k\nu_{A\to B}}{N}‐\frac{k\alpha \nu_{A\to B}}{N^{2}}+\frac{\nu_{B\to A}}{N}\left(N‐k\right)\right)}{N+\frac{\alpha k}{N}}=x+O\left(\frac{1}{N}\right).$$

Therefore, the infinitesimal variance for *N* → ∞ is given by

(62) $$\sigma^{2}\left(x\right)=x\left(1‐x\right).$$

Putting together the final results for the infinitesimal mean and variance, we get the weak selection and mutation limit of the Wright–Fisher model with selection and mutation, that is,

(63) $$dx_{t}=\left(\alpha x_{t}\left(1‐x_{t}\right)‐\nu_{A\to B}x_{t}+\nu_{B\to A}\left(1‐x_{t}\right)\right)dt+\sqrt{x_{t}\left(1‐x_{t}\right)}dW_{t}.$$

## APPENDIX 3

### FREQUENCY‐DEPENDENT SELECTION

In the main text, we have exclusively considered situations with constant selection coefficients *s* (or *α*). Here instead, we apply the derived formulas for the fixation probability and the mean time to fixation for the case of frequency‐dependent selection. More precisely, we consider a stochastic diffusion with linear frequency dependence (we refer to Traulsen et al. (2006) for a physical formulation of this and Pfaffelhuber and Wakolbinger (2018) for a more general mathematical analysis). We denote the strength of selection by *α* and let *u*, *v* be arbitrary real numbers. We write *αx*(1 − *x*)(*ux* + *v*) for the linear frequency‐dependent dynamics of selection. Then, the allele frequency evolves according to the following equation

(64) $$dx_{t}=\alpha x_{t}\left(1‐x_{t}\right)\left(ux_{t}+v\right)dt+\sqrt{x_{t}\left(1‐x_{t}\right)}dW_{t}.$$

We have *μ*(*x*) = *αx*(1 − *x*)(*ux* + *v*) and *σ* ^2^(*x*) = *x*(1 − *x*).

#### Fixation probability

Recall the formula for the fixation probability, Equation 42

(65) $$P_{x_{0}}\left(x_{\infty }=1\right)=\frac{S\left(x_{0}\right)‐S\left(0\right)}{S(1)‐S(0)},$$

where *S*(*x*) is the scale function given in Equation 32 and given by

(66) $$S(x)=\begin{array}{c} \int_{}^{x} \end{array}\exp\left(‐2\begin{array}{c} \int_{}^{y} \end{array}\frac{\mu \left(z\right)}{\sigma^{2}\left(z\right)}dz\right)dy,\;x\in (0,1).$$

For *α* = 1, we can linearize the exponential and write the scale function as

(67) $$\begin{array}{cc} S(x) & =\int_{}^{x}\exp\left(‐2\int_{}^{y}y\alpha (v+uz)dz\right)dy\\ & =\int_{}^{x}\exp(‐2\alpha vy‐\alpha uy^{2})dy\\ & \overset{\alpha \ll 1}{\approx }x‐2\alpha \int_{}^{x}\left(vy+\frac{1}{2}uy^{2}\right)dy\\ & =x‐\alpha vx^{2}‐\frac{\alpha }{3}ux^{3}. \end{array}$$

Plugging this into Equation 65, we obtain

(68) $$\begin{array}{cc} \frac{S\left(x\right)‐S\left(0\right)}{S\left(1\right)‐S\left(0\right)} & =\frac{S\left(x\right)}{S\left(1\right)}=\frac{x\left(1‐\alpha vx‐\frac{\alpha }{3}ux^{2}\right)}{1‐\alpha v‐\frac{\alpha }{3}u}\\ & =x\left(1‐\alpha vx‐\frac{\alpha }{3}ux^{2}+\alpha v+\frac{\alpha }{3}u\right)+O\left(\alpha^{2}\right)\\ & =x\left(1+\alpha u\left(\frac{v}{u}\left(1‐x\right)+\frac{1}{3}\left(1‐x^{2}\right)\right)\right)+O\left(\alpha^{2}\right). \end{array}$$

In the context of evolutionary game theory, this result is a rederivation of the 1/3‐law (Nowak et al., 2004) (generalized by Lessard & Ladret, 2007). It states that for an allele starting with one individual, it is more likely to become fixed in the population than under neutral dynamics if the deterministic fixed point is smaller than 1/3. This can be seen by plugging in *u* = *a* − *b* − *c* + *d* and *v* = *b* − *d*, where *a*, *b*, *c*, and *d* represent the payoffs of an evolutionary game.

#### Mean time to fixation

The mean time to fixation is given by Equation 46 that was given as

(69) $$\begin{array}{cc} E_{x}\left[\tau \right] & =\int_{0}^{1}G(x,y)dy\\ & =\int_{0}^{x}2\frac{\left(S(1)‐S(x)\right)}{\left(S(1)‐S(0)\right)}\frac{\left(S(y)‐S(0)\right)}{\sigma^{2}\left(y\right)S^{\prime }\left(y\right)}dy+\int_{x}^{1}2\frac{\left(S(x)‐S(0)\right)}{\left(S(1)‐S(0)\right)}\frac{\left(S(1)‐S(y)\right)}{\sigma^{2}\left(y\right)S^{\prime }\left(y\right)}dy, \end{array}$$

where *G*(*x*,*y*) is Green's function and defined as (Equation 45)

(70) $$G\left(x,y\right)=\left\{\begin{array}{c} 2\frac{S(x)‐S(0)}{S(1)‐S(0)}\left(S\left(1\right)‐S\left(y\right)\right)m\left(y\right),\;0\leq x\leq y\leq 1,\\ 2\frac{S(1)‐S(x)}{S(1)‐S(0)}\left(S\left(y\right)‐S\left(0\right)\right)m\left(y\right),\;0\leq y\leq x\leq 1, \end{array}\right)$$

Similar to the computation of the fixation probability, we will consider the case of small initial frequencies and weak selection, that is, *α*,*x* << 1. More precisely, we neglect terms of order *α* ^2^ and *αx* ^2^. We recall the approximation of the scale function in this case that we derived in Equation 67

(71) $$S(x)\overset{\alpha \ll 1}{\approx }x‐\alpha vx^{2}‐\frac{\alpha }{3}ux^{3}\overset{x\ll 1}{\approx }x.$$

Employing these approximations, the first integral in Equation 69 yields

(72) $$\begin{array}{c} \int_{0}^{x}\left[2\frac{\left(S(1)‐S(x)\right)}{S(1)‐S(0)}\frac{\left(S(y)‐S(0)\right)}{\sigma^{2}\left(y\right)S^{\prime }\left(y\right)}\right]dy\\ \;=2\frac{S(1)‐S(x)}{S(1)}\int_{0}^{x}\left[\frac{y\left(1‐\alpha vy‐\frac{\alpha }{3}uy^{2}\right)}{y\left(1‐y\right)\left(1‐2\alpha vy‐\alpha uy^{2}\right)}\right]dy\\ \;\approx 2\left(1‐\frac{x}{\left(1‐\alpha v‐\alpha \frac{u}{3}\right)}\right)\int_{0}^{x}\left[\frac{\left(1‐\alpha \left(vy+\frac{u}{3}y^{2}\right)\right)\left(1+\alpha \left(2vy+uy^{2}\right)\right)}{\left(1‐y\right)}\right]dy\\ \;\approx 2\left(1‐x\left(1+\alpha \left(v+\frac{u}{3}\right)\right)\right)\int_{0}^{x}\left[\frac{1‐\alpha \left(vy+\frac{u}{3}y^{2}‐2vy‐uy^{2}\right)}{\left(1‐y\right)}\right]dy\\ \;\approx 2\left(1‐x\right)\left(\int_{0}^{x}\frac{1}{1‐y}dy+\underset{\in O(\alpha x^{2})}{\underbrace{\int_{0}^{x}\frac{\alpha y\left(v+\frac{2}{3}y\right)}{1‐y}dy}}\right)\\ \;\approx 2\left(1‐x\right)\ln\left(1‐{\left(1‐x\right)}^{‐1}\right). \end{array}$$

Approximating the second integral in a similar way, we find

$$\begin{array}{c} \int_{x}^{1}2\frac{S(x)}{S(1)}\frac{\left(S(1)‐S(y)\right)}{\sigma^{2}\left(y\right)S\prime \left(y\right)}dy\\ \;=2S\left(x\right)\int_{x}^{1}\left[\left(1‐\frac{S(y)}{S(1)}\right)\frac{1}{y\left(1‐y\right)\left(1‐2\alpha vy‐\alpha uy^{2}\right)}\right]dy\\ \;\approx 2S\left(x\right)\int_{x}^{1}\left[\left(1‐\frac{y\left(1‐\alpha vy‐\frac{\alpha }{3}uy^{2}\right)}{1‐\alpha \left(v+\frac{u}{3}\right)}\right)\frac{\left(1+\alpha \left(2vy+uy^{2}\right)\right)}{y(1‐y)}\right]dy\\ \;\approx 2S\left(x\right)\int_{x}^{1}\left[\left(1‐y\left(1‐\alpha vy‐\frac{\alpha }{3}uy^{2}\right)\left(1+\alpha \left(v+\frac{u}{3}\right)\right)\right)\frac{\left(1+\alpha y(2v+uy)\right)}{y(1‐y)}\right]dy\\ \;\approx 2S\left(x\right)\int_{x}^{1}\left[\left(1‐y‐\alpha y\left(v\left(1‐y\right)‐\frac{u}{3}\left(1‐y^{2}\right)\right)\right)\frac{\left(1+\alpha y(2v+uy)\right)}{y(1‐y)}\right]dy\\ \;\approx 2S\left(x\right)\int_{x}^{1}\left[\frac{1}{y}+\alpha \left(2v+uy\right)‐\frac{\alpha \left(v\left(1‐y\right)+\frac{u}{3}\left(1‐y^{2}\right)\right)}{1‐y}\right]dy\\ \;=2S\left(x\right)\int_{x}^{1}\left[\frac{1}{y}+\alpha \left(2v+uy‐v‐\frac{u}{3}\left(1+y\right)\right)\right]dy\\ \;\approx 2x\ln\left(x^{‐1}\right)+2x\alpha \left(v\left(1‐x\right)+\frac{u}{3}\left(1‐x^{2}\right)‐\frac{u}{3}\left(1‐x\right)\right)\\ \;\approx 2x\ln(x^{‐1})+2x\alpha v. \end{array}$$

Taking these two expressions together we rederived the results already known in the literature (Altrock & Traulsen, 2009; Pfaffelhuber & Wakolbinger, 2018), that is,

(74) $$E_{x}\left[\tau \right]\approx 2\left(1‐x\right)\ln\left({\left(1‐x\right)}^{‐1}\right)+2x\ln\left(x^{‐1}\right)+2x\alpha v,$$

for *α* and *x* sufficiently small.
